# Weather, day length and physical activity in older adults: Cross-sectional results from the European Prospective Investigation into Cancer and Nutrition (EPIC) Norfolk Cohort

**DOI:** 10.1371/journal.pone.0177767

**Published:** 2017-05-31

**Authors:** Yu-Tzu Wu, Robert Luben, Nicholas Wareham, Simon Griffin, Andy P. Jones

**Affiliations:** 1 Department of Population Health & Primary Care, Norwich Medical School, University of East Anglia, Norwich, United Kingdom; 2 UKCRC Centre for Diet and Activity Research (CEDAR), Institute of Public Health, University of Cambridge, Forvie Site, Robinson Way, Cambridge, United Kingdom; 3 Department of Public Health and Primary Care, Institute of Public Health, University of Cambridge, Cambridge, United Kingdom; Kent State University, UNITED STATES

## Abstract

**Background:**

A wide range of environmental factors have been related to active ageing, but few studies have explored the impact of weather and day length on physical activity in older adults. We investigate the cross-sectional association between weather conditions, day length and activity in older adults using a population-based cohort in England, the European Prospective Investigation into Cancer and Nutrition (EPIC) Norfolk study.

**Methods:**

Physical activity was measured objectively over 7 days using an accelerometer and this was used to calculate daily total physical activity (counts per minute), daily minutes of sedentary behaviour and light, moderate and vigorous physical activity (LMVPA). Day length and two types of weather conditions, precipitation and temperature, were obtained from a local weather station. The association between these variables and physical activity was examined by multilevel first-order autoregressive modelling.

**Results:**

After adjusting for individual factors, short day length and poor weather conditions, including high precipitation and low temperatures, were associated with up to 10% lower average physical activity (p<0.01) and 8 minutes less time spent in LMVPA but 15 minutes more sedentary time, compared to the best conditions.

**Conclusion:**

Day length and weather conditions appear to be an important factor related to active ageing. Future work should focus on developing potential interventions to reduce their impact on physical activity behaviours in older adults.

## Introduction

There has been particular interest in the potential for the environment to support active ageing, the process of optimising opportunities for health and well-being as individuals grow older [1]. A wide range of environmental factors have been related to physical activity in older adults [2,3]. Although the idea of age-friendly environments has been promoted worldwide, projects have mainly focused on the characteristics of the built and social environment [4]. Nevertheless other environmental conditions that are out of direct control of planners, such as weather conditions and day length, may interact with features of these environments to influence individual activity levels [5] and might thus have an impact on active ageing [6].

Literature in the field of environmental gerontology has proposed the ‘Environmental Press Model’, suggesting that adults with reduced individual competence, such as the aged, are more sensitive to stress from the environment and that this may lead to maladaptive behaviours and poor health [7]. Adverse weather conditions, such as heavy rain, low temperatures and short daylight hours, could be one potential source of environmental stress. Older adults, who are more likely to experience functional and health declines, might be especially sensitive to poor weather conditions, which have been reported to have a detrimental influence on physical activity in younger age groups [5,8,9]. In the elderly there is evidence from qualitative studies that weather and seasonal factors may be associated with concerns such as poor visibility and slippery surfaces [10–12]. However, there is a lack of empirical evidence on the nature and magnitude of associations.

Only a small amount of research has used objective measures of physical activity to investigate associations with weather, climate and physical activity in older adults [13–15]. Amongst 1324 German older adults, Klenk et al [14] showed linear relationships between the duration of walking and a wide range of weather variables including daylight, maximum temperature, total global radiation, average precipitation, average wind speed and average humidity. In a rural Scottish study of 548 adults, Witham et al [13] explored potential effect modifiers on the association between weather conditions and accelerometer derived activity levels and found higher daily temperature and longer day length were associated with higher activity levels. More recently, Prins & van Lenthe [15] used a GPS logger to determine associations between hourly weather conditions and walking and cycling behaviour among 43 older adults in the Netherlands, reporting a positive relationship between hourly temperature, walking and cycling minutes per hour.

Whilst these recent studies have given new insights into the potential role of weather as a determinant of physical activity in older adults, they have a number of limitations. One is that none took into account temporal autocorrelation when examining the association between weather and physical activity. This is a key methodological limitation because daily trends in both weather and physical activity are like to follow a temporally autocorrelated time series pattern whereby observations for one day are likely to be associated with those on the subsequent day. Failure to account for this in model specification leads to biases in model results [16]. Further, none of the studies examined different intensities of activity and in particular none looked at sedentary behaviour. Time spent sedentary has been particularly related to physical function, disability and metabolic syndrome in older age [17,18].

Using appropriate statistical methodologies for the analysis for time-series data, this study explores associations between weather conditions, day length and physical activity amongst large well-characterised population of older adults. The analysis is based on a population-based cohort in England: the European Prospective Investigation into Cancer and Nutrition (EPIC) Norfolk study, which collected objective measures of physical activity in over 4000 older adults between 2006 and 2011.

## Materials and methods

### Study population

The European Prospective Investigation into Cancer and Nutrition (EPIC) Norfolk study is one of population-based cohorts from the 10-county collaboration of the European Prospective Investigation into Cancer and Nutrition (EPIC), which was originally designed to examine the associations between diet and cancer. The scope of data collection has since been expanded to investigate major determinants of chronic disease, disability and death in middle and later life [19].

Details of the EPIC sampling and recruitment have been described elsewhere [20]. Briefly, EPIC Norfolk participants were recruited at baseline aged 45–74 between 1993 and 1997 from general practices across the county of Norfolk. In total 77630 individuals were invited and 30445 consented to take part. At the third health check, between September 2006 and December 2011, 8623 attended a health examination. Of these 4207 wore an accelerometer to measure their physical activity. The EPIC Norfolk study was approved by the Norfolk Local Research Ethics Committee (05/Q0101/191) and East Norfolk and Waveney National Health Service Research Governance Committee (2005EC07L) and written consent was obtained from participants [19]. This secondary data analysis does not require new IRB approval.

### Measurement of physical activity

Physical activity was measured using a commercial accelerometer (Actigraph GT1M, Florida USA), which was set to a 5 second epoch. The EPIC Norfolk participants attending the third health check were invited to wear the accelerometer to measure their daily physical activity. Those who agreed to take part were instructed to wear the equipment for seven continuous days. Valid days were defined as those with evidence that the accelerometer was worn for at least 10 hours after screening out period of non-wear time, which was defined as continuous zero strings of ≥90 minutes duration. Participants with less than four valid days were excluded from the analysis. After excluding non-valid days and those with insufficient data, a total of 27446 person days of accelerometery were available for this research. The mean wear time was 869 (SD: 95) minutes per day in spring, 875 (SD: 89) in summer, 869 (SD: 99) in autumn and 865 (SD: 96) in winter.

Three types of physical activity measures were generated for each participant day using the accelerometer data. Mean daily counts per minute, a summarised indicator of daily activity level, were calculated using the total daily counts as recorded by the Actigraph divided by total wear minutes. Sedentary behaviour was defined by valid periods below 100 counts per minutes. As older adults are typically not vigorously active, the analysis presented here focused on light, moderate and vigorous physical activity (LMVPA) as opposed to the commonly employed moderate to vigorous activity (MVPA). LMVPA, which was defined as that over 1000 accelerometery counts per minute, includes any activities ranging from slow walking to vigorous exercise.

### Environmental conditions: Day length and weather

Day length, precipitation and temperature have previously been suggested to be related to physical activity in older adults [13,14]. Hourly measurements of temperature and precipitation data were obtained from the Marham Norfolk weather station, which was the closest to the study area. Data from the weather station was obtained for each day during the study period, and used to calculate daily cumulative precipitation (mm) from 6am to 10pm and identify the maximum and minimum daytime temperature (Celsius) for the study period. In addition, day length (hours) was computed based on an algorithm that used latitude [21].

Trends across the variables were examined by classifying them into categories. Since a large number of days had no precipitation, days without rain (i.e. 0mm) were grouped into one category and those with some rain were divided into non-zero tertiles. The other three measures, maximum and minimum daytime temperature and day length, were categorised into quartiles.

### Covariates

Demographic information on gender and education was collected at the baseline. Education was divided into four levels: no education, O-level (10–11 years), A-level (12–13 years) and university degree or equivalent. Since adults with poor health tend to have lower level of physical activity, measures of self-rated health were obtained from the third health check questionnaires. Self-rated health was measured by the question “How would you rate your general health?”. Adults reporting excellent, very good and good health were categorised into one group and those reporting fair and poor were in the other group. This single question has been widely used in health research [22] and has also been recognised as a predictor of mortality [23].

### Statistical analysis

The association between daily physical activity and daily weather conditions in the cohort was examined using regression models fitted a two level multilevel structure of days nested within individuals. Between days, the association between weather and physical activity was anticipated to exhibit temporal autocorrelation and hence multilevel first-order autoregressive modelling was employed [24].

Three types of models were fitted to the three measures of daily counts per minutes, sedentary time and LMVPA time. First, unadjusted associations between physical activity and weather conditions were examined, and then these were adjusted for individual level factors including age, gender, education and self-rated health. Finally, a full model including both individual level factors and weather conditions was fitted to investigate the independent association between weather conditions and physical activity. As variation in accelerometer wear time is likely to cause differences in recorded physical activity, daily minutes of time participants wore the accelerometers was added as a covariate for the models of sedentary behaviour and LMVPA. A significance level of p<0.05 was used in this study.

## Results

Table 1 shows descriptive characteristics of those 4051 participants with at least four valid days of physical activity data. The mean age was 69.0yrs with a range from 49 to 92yrs. The cohort was relatively well educated; almost 65% of participants had an A-level education or better. Under 15% of participants reported fair or poor health.

**Table 1:** Distributions of demographic factors and health status in the study sample (N,%).

	Men N = 1796	Women N = 2255	Total N = 4051
Age group			
<65	529 (29.5)	891 (39.5)	1420 (35.1)
65–69	397 (22.1)	504 (22.4)	901 (22.2)
70–74	392 (21.8)	413 (18.3)	805 (19.9)
75–79	273 (15.2)	282 (12.5)	555 (13.7)
80+	205 (11.4)	165 0(7.3)	370 0(9.1)
Education (missing = 1)			
No education	358 (19.9)	668 (29.6)	1026 (25.4)
O level	190 (10.6)	296 (13.1)	486 (12.0)
A level	892 (49.7)	942 (41.8)	1834 (45.3)
Degree	355 (19.8)	349 (15.5)	704 (17.4)
Self-reported health (missing = 94)			
Excellent/very good/good	1481 (84.3)	1895 (86.1)	3376 (85.3)
Fair/poor	275 (15.7)	306 (13.9)	581 (14.7)

The mean of daily counts per minute recorded was 256.1 (SD: 150.6) with a range from 3.8 to 1744.8. Mean recorded minutes of daily sedentary behaviour was high at nearly 679 mins (SD: 101.8), which equates to about 11 hours. The mean time spent in LMVPA per day was 73 mins (SD: 43.4) with a maximum of 374.4 mins recorded by one participant. Older age, being female, lower education and poorer self-rated health were generally associated with lower level of physical activity and increased daily sedentary time (Table 2).

**Table 2:** The associations between physical activity and demographic factors.

	Daily counts per minutes (counts)		Sedentary behaviour (minutes/day)		Light, moderate and vigorous physical activity (minutes/day)	
	Unadjusted	Adjusted^1^	Unadjusted	Adjusted^1^	Unadjusted	Adjusted^1^
Age						
<65 (ref.)	-	-	-	-	-	-
65–69	-29.4 (-38.4, -20.5)	-30.5 (-39.4, -21.7)	11.3 (6.9, 15.7)	10.9 (6.5, 15.2)	-7.7 (-10.3, -5.1)	-8.0 (-10.5, -5.4)
70–74	-60.4 (-69.6, -51.1)	-58.1 (-67.3, -48.9)	19.6 (15.0, 24.1)	17.4 (12.9, 21.9)	-15.8 (-18.4, -13.1)	-15.3 (-17.9, -12.6)
75–79	-105.8 (-116.2, -95.1)	-104.1 (-114.5, -93.6)	40.1 (34.9, 45.3)	37.9 (32.8, 43.0)	-29.3 (-32.3, -26.3)	-28.9 (-31.9, -25.9)
80+	-160.8 (-173.0, 148.5)	-157.0 (-169.2, -144.7)	67.1 (61.1, 73.2)	63.0 (57.0, 69.1)	-45.9 (-49.4, -42.4)	-45.0 (-48.5, -41.5)
p.^2^	<0.001	<0.001	<0.001	<0.001	<0.001	<0.001
Sex						
Men (ref.)	-	-	-	-	-	-
Women	-0.7 (-8.1, 6.6)	-12.0 (-18.7, -5.4)	-18.6 (-22.1, -15.2)	-13.8 (-17.0, -10.5)	0.9 (-1.2, 3.0)	-2.5 (-4.4, -0.6)
p.^2^	0.85	<0.001	<0.001	<0.001	0.42	0.01
Education						
Degree (ref.)	-	-	-	-	-	-
A-level	-19.0 (-29.2, -8.8)	-13.2 (-22.3, -4.1)	-2.2 (-7.1, 2.7)	-4.1 (-8.6, 0.4)	-3.3 (-6.2, -0.3)	-1.7 (-4.3, 1.0)
O-level	-22.4 (-36.0, -8.8)	-22.8 (-35.0, -10.6)	-1.8 (-8.3, 4.8)	0.4 (-5.6, 6.4)	-3.9 (-7.8, -0.1)	-4.2 (-7.7, -0.7)
None	-37.5 (-48.8, -26.2)	-14.7 (-24.9, -4.4)	-1.8 (-7.2, -3.6)	-7.6 (-12.6, -2.6)	-7.2 (-10.5, -4.0)	-1.1 (-4.1, 1.8)
p.^2^	<0.001	0.002	0.86	0.006	<0.001	0.12
Self-rated health						
Good/excellent (ref.)	-	-	-	-	-	-
Fair/poor	-69.7 (-79.8, -59.5)	-56.9 (-66.2, -47.7)	29.4 (24.5, 34.3)	24.5 (20.0, 29.1)	-19.3 (-22.2, -16.4)	-15.8 (-18.5, -13.1)
p.^2^	<0.001	<0.001	<0.001	<0.001	<0.001	<0.001

^1.^ The adjusted model included all the variables.

^2.^ p-value of test for heterogeneity.

**Fig 1:**
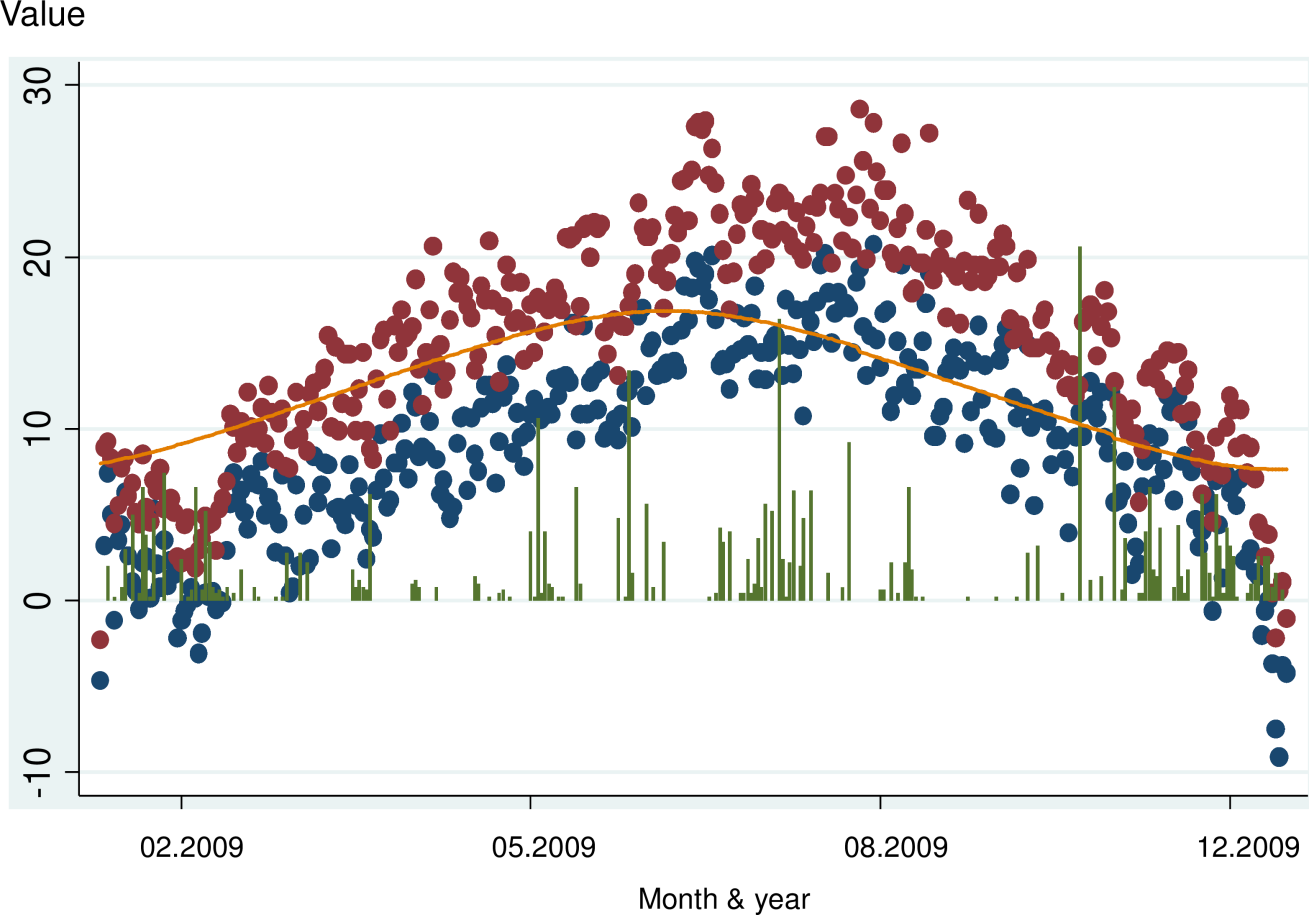
Changes in environmental conditions over the period of the study (red/blue: Maximum/Minimum temperature (°C); green: precipitation (mm); orange: day length (hour)).

Precipitation, temperature and day length over the period of investigation are charted in Fig 1. Daily precipitation ranged from 0 to 26.2 mm with 54% of days being totally dry. The mean maximum and minimum daytime temperature in the study areas was 14.3 and 8.6 degrees Celsius with these two measures being highly correlated (r = 0.93, p<0.001). Day length ranged from 7.6 to 16.9 hours.

### Weather conditions, day length and physical activity

**Fig 2:**
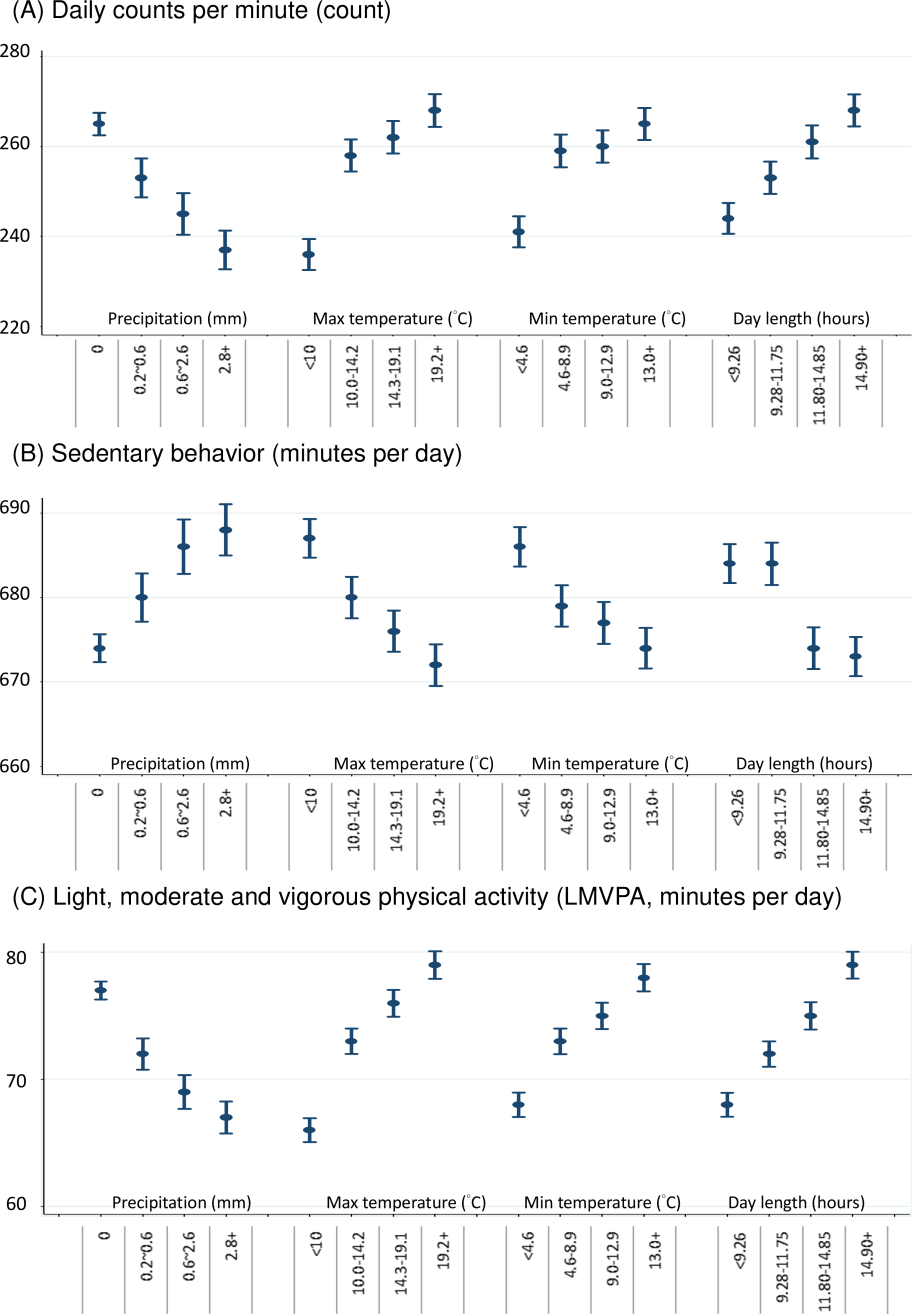
Mean of physical activity measures by different environmental conditions.

Fig 2 depicts mean values of the physical activity measures by different conditions examined. Daily counts per minute and LMVPA were higher with higher minimum and maximum temperature and day length and were lower with higher levels of daily precipitation. Daily LMVPA showed similar patterns to daily counts per minutes. Minutes of sedentary behaviour were higher with higher precipitation and lower with higher temperature and longer day length.

Table 3 reports both unadjusted and adjusted associations between physical activity and weather conditions. Significant associations remained with all measures of environmental conditions after adjusting for individual level factors. Daily counts per minute were 26.0 (95% CI: -29.9, -22.0) lower in days with the greatest precipitation (>2.8 mm) compared to dry days. Similar trends in daily counts per minute were observed across the quartile groups for maximum temperature (-29.1; 95% CI: -35.3, -22.9) and day length (-25.9; 95% CI: -34.4, -17.4). On days in the highest precipitation quartile, sedentary time was nearly 15 minutes higher than dry days (14.4; 95% CI: 12.7, 16.2) and time spent in LMVPA time was 8 minutes lower (-8.2; 95% CI: -9.3, -7.1). On the coldest days (maximum temperature <10.0 Celsius), time spent sedentary was nearly 20 minutes higher (19.2; 95% CI: 16.4, 22.0) and LMVPA time by 10 minutes lower (-10.8, 95% CI: -12.6, -9.1) than days with a maximum temperature of over 19 Celsius, although the difference across the quartile groups for minimum temperature was smaller. There was higher sedentary time (20.7; 95% CI: 16.6, 24.8) recorded on the shortest days (<9.3 hours) along with less time spent in LMVPA (-10.0; 95% CI: -12.5, -7.6).

**Table 3:** The associations between physical activity, day length and weather.

		Daily counts per minutes		Sedentary behaviour (min/day)		LMVPA (min/day)	
		Unadjusted	Adjusted^1^	Unadjusted	Adjusted^1^	Unadjusted	Adjusted^1^
		(N = 27446)	(N = 26805)	(N = 27446)	(N = 26805)	(N = 27446)	(N = 26805)
Precipitation	0 mm (ref)	-	-	-	-	-	-
	0.2~0.6	-10.6 (-14.3, -6.9)	-10.6 (-14.3, -6.9)	6.1 (4.5, 7.7)	6.9 (4.5, 7.7)	-3.4 (-4.4, -2.4)	-3.4 (-4.4, -2.4)
	0.6~2.6	-17.6 (-21.5, -13.6)	-17.7 (-21.7, -13.7)	9.7 (8.0, 11.5)	9.9 (8.1, 11.6)	-5.7 (-6.8, -4.6)	-5.8 (-6.9, -4.7)
	2.8+	-26.3 (-30.2, -22.3)	-26.0 (-29.9, -22.0)	14.5 (12.8, 16.2)	14.4 (12.7, 16.2)	-8.3 (-9.3, -7.2)	-8.2 (-9.3, -7.1)
	p.^2^	<0.001	<0.001	<0.001	<0.001	<0.001	<0.001
Max temperature	19.2+ °C (ref)	-	-	-	-	-	-
	14.3–19.1	-9.2 (-13.6, -4.8)	-9.2 (-13.6, -4.9)	7.0 (5.0, 8.9)	7.0 (5.0, 8.9)	-3.8 (-5.0, -2.6)	-3.8 (-5.0, -2.6)
	10.0–14.2	-16.5 (-22.1, -10.8)	-17.1 (-22.7, -11.6)	12.6 (10.1, 15.1)	12.6 (10.1, 15.1)	-6.6 (-8.1, -5.0)	-6.9 (-8.4, -5.3)
	<10	-28.7 (-35.1, -22.3)	-29.1 (-35.3, -22.9)	19.2 (16.4, 22.0)	19.2 (16.4, 22.0)	-10.6 (-12.4, -8.8)	-10.8 (-12.6, -9.1)
	p.^2^	<0.001	<0.001	<0.001	<0.001	<0.001	<0.001
Min temperature	13.0+ °C (ref)	-	-	-	-	-	-
	9.0–12.9	-5.8 (-10.3, -1.3)	-6.1 (-10.6, -1.6)	5.1 (3.1, 7.1)	5.1 (3.1, 7.1)	-2.7 (-3.9, -1.4)	-2.7 (-4.0, -1.5)
	4.6–8.9	-6.7 (-12.3, -1.2)	-7.6 (-13.1, -2.2)	7.5 (5.0, 10.0)	7.8 (5.4, 10.3)	-3.7 (-5.2, -2.2)	-4.0 (-5.6, -2.5)
	<4.6	-11.8 (-18.0, -5.7)	-13.2 (-19.1, -7.2)	9.8 (7.1, 12.6)	10.6 (7.9, 13.3)	-5.2 (-6.9, -3.5)	-5.6 (-7.3, -4.0)
	p.^2^	<0.001	<0.001	<0.001	<0.001	<0.001	<0.001
Day length	14.90+ hr (ref)	-	-	-	-	-	-
	11.80–14.85	-5.9 (-14.7, 2.9)	-7.0 (-15.1, 1.0)	6.2 (2.2, 10.3)	6.9 (3.0, 10.7)	-2.6 (-5.0, -0.1)	-3.0 (-5.3, -0.7)
	9.28–11.75	-13.6 (-22.8, -4.5)	-17.6 (-26.0, -9.3)	14.6 (10.4, 18.9)	16.4 (12.5, 20.4)	-6.2 (-8.7, -3.6)	-7.4 (-9.8, -5.0)
	<9.26	-22.1 (-31.5, -12.6)	-25.9 (-34.4, -17.4)	19.3 (14.9, 23.7)	20.7 (16.6, 24.8)	-8.8 (-11.5, -6.1)	-10.0 (-12.5, -7.6)
	p.^2^	<0.001	<0.001	<0.001	<0.001	<0.001	<0.001

^1.^ Adjusted for age, gender, education and self-rated health

^2.^ p.: p-value of test for trend

The models in Table 4 include all individual level factors and three measures of environmental conditions: precipitation, maximum temperature and day length together. Since maximum and minimum temperatures were strongly correlated, this fully adjusted model only included maximum temperature which generally had a lager effect size than minimum temperature. The three measures were still significantly associated with physical activity jointly although the effect sizes for maximum temperature and day length were attenuated (by 40~50% in the highest quartile) compared to that observed before joint adjustment.

**Table 4:** Fully adjusted models showing associations between physical activity and joint environmental conditions.

	Daily counts per minutes^1^	Sedentary behaviour (minutes/day) ^1^	LMVPA (minutes/day)^1^
Precipitation			
0 mm (ref)	-	-	-
0.2~0.6	-9.1 (-12.8, -5.4)	5.3 (3.7, 6.9)	-2.9 (-3.9, -1.8)
0.6~2.6	-16.1 (-20.1, -12.0)	8.8 (7.1, 10.6)	-5.2 (-6.3, -4.0)
2.8+	-24.7 (-28.7, -20.7)	13.6 (11.9, 15.3)	-7.7 (-8.8, -6.6)
p.^2^	<0.001	<0.001	<0.001
Max temperature			
>19.1°C (ref)	-	-	-
14.3–19.1	-5.3 (-9.8, -0.9)	4.3 (2.3, 6.2)	-2.4 (-3.7, -1.2)
10.0–14.2	-8.5 (-14.7, -2.3)	6.5 (3.7, 9.2)	-3.8 (-5.6, -2.1)
<10.0	-18.8 (-26.0, -11.5)	11.7 (8.5, 14.9)	-7.2 (-9.2, -5.2)
p. ^2^	<0.001	<0.001	<0.01
Day length			
>14.85 hrs (ref)	-	-	-
11.80–14.85	-4.6 (-12.7, 3.6)	5.2 (1.3, 9.0)	-2.0 (-4.3, 0.4)
9.28–11.75	-9.2 (-18.2, -0.3)	11.3 (6.9, 15.3)	-4.1 (-6.7, -1.6)
<9.26	-12.5 (-22.3, -2.7)	12.7 (8.1, 17.3)	-5.0 (-7.8, -2.3)
p. ^2^	0.02	<0.001	<0.001

^1.^ First-order autoregressive models included all individual (age, gender, education and self-rated health) and weather factors

^2.^ p.: p-value of test for trend

## Discussion

### Main findings

This study investigated the association between day length and weather conditions (precipitation and temperature), physical activity (daily counts per minute and LMVPA time) and sedentary behaviour in older English audits. Short day length and poorer weather conditions, particularly heavy rain and lower temperatures, were associated with up to a 10% reduction in physical activity (25 counts per minute per day or 8 minutes of LMVPA) and a 2% more time sedentary (corresponding to 15 minutes) compared to the average of the whole study population. The associations between day length, weather conditions and physical activity were largely independent of individual level factors and were attenuated but remained after joint adjustment.

### Strengths and limitations

Strengths of the study include the fact that it was based on a large population-based cohort of older English adults with objective measures of physical activity for seven days. Objectively measured physical activity can improve limitations of self-reported data and reduce potential recall bias. Unlike previous studies [13–15], this study used multilevel time-series modelling to take into account two-level data structure as well as temporal autocorrelation inherent in this type of data.

In terms of limitations, the study population lived in Norfolk, an area situated in East of England. Although daily weather changed throughout the year, the overall climate of this area is mild with less extreme weather conditions compared to some regions in England or other countries. The impact of day length and weather on daily activity could thus be larger in localities with more extreme conditions. Although the literature has suggested that older people are more active in the morning than afternoon or evening [25], this study did not explore the relationship between physical activity and hourly weather conditions as only a single weather station provided data. Although the Marham is only located 50 km from Norwich, the largest city in Norfolk, hourly weather data may not have been representative of the whole study area at any point in time.

This analysis only used a single question to measure health status and did not include complete information from medical records or health examinations. However, self-rated health has been related to mortality and can provide valid insight into individual health in general [23]. A relatively small proportion (15%) of participants reported fair or poor health and this suggests there may have been some selection bias whereby healthier individuals were more likely to remain in the cohort. In common with most other studies, we chose a 7-day wear period for the accelerometer. However, it is possible that patterns of habitual physical activity may not be completely captured over a monitoring period of this length.

### Physical activity, sedentary behaviour and weather conditions

The findings of this study show that weather conditions were independently associated with physical activity in older adults. Our results correspond to those of previous studies in other settings variation in climate and other local factors [13–15]. We found heavier rain, lower temperatures and shorter day length were associated with lower physical activity. Although we were unable to differentiate time spent indoors and outdoors in this work, we suspect these conditions might strongly affect outdoor activity, which has been shown to substantially contribute to daily activity level in older age [25]. Findings from qualitative research suggest that concerns over safety, fear of falling and injury are potential barriers to outdoor activity in older adults [26,27]. Poor weather conditions may hence increase these worries and lead to reduction in outdoor activity in older adults.

In this older population, much of the time each day was spent sedentary (on average around 11 hours). Sedentary behaviour has been suggested to be related to poor health and act as a risk factor for mortality, cardiovascular diseases and metabolic syndrome [28,29]. A recent meta-analysis evaluating interventions to reduce sedentary time showed a mean reduction of 22 minutes per day among 51 studies [30]. In this study, we observed nearly 15 minutes higher sedentary time on a day with poorest weather compared to best; rather similar to the effect of the reviewed interventions focusing on individual lifestyle and behaviour factors. This suggests that alleviating the negative influence of poor weather may be a possible direction for public health interventions in older adults.

### Future research directions and public health implications

To support active ageing and develop possible interventions, future research may explore the mechanism by which weather acts as a determinant of physical activity. For example, since older adults may be more hesitant to leave home in poor conditions due to safety concerns [26,27], a potential intervention could be to improve outdoor environments to be more resilient to poor weather such as adding anti-slip surfaces for pavements or lighting in certain areas. An alternative approach could be to increase individual competence to maintain activity level in days with poor weather. Improving clothing and equipment for wet weather might address some concerns in older adults, whilst enhancing motivation for physical activity could be another direction. An example could be encouraging dog ownership where appropriate, as this has been suggested to help protect against declines in physical activity during periods of poor weather [31].

## Supporting information

S1 Checklist STROBE checklist. (DOC)
